# Finding a Balance between Protection and Pathology: The Dual Role of Perforin in Human Disease

**DOI:** 10.3390/ijms18081608

**Published:** 2017-07-25

**Authors:** Robin C. Willenbring, Aaron J. Johnson

**Affiliations:** 1 Mayo Clinic Graduate School of Biomedical Sciences, Rochester, MN 55905, USA; Willenbring.robin@mayo.edu; 2 Department of Immunology, Mayo Clinic, Rochester, MN 55905, USA

**Keywords:** perforin, familial hemophagocytic lymphohistiocytosis type 2, blood–brain barrier disruption, single nucleotide variants, selective advantage

## Abstract

Perforin is critical for controlling viral infection and tumor surveillance. Clinically, mutations in perforin are viewed as unfavorable, as lack of this pore-forming protein results in lethal, childhood disease, familial hemophagocytic lymphohistiocytosis type 2 (FHL 2). However, many mutations in the coding region of *PRF1* are not yet associated with disease. Animal models of viral-associated blood–brain barrier (BBB) disruption and experimental cerebral malaria (ECM) have identified perforin as critical for inducing pathologic central nervous system CNS vascular permeability. This review focuses on the role of perforin in both protecting and promoting human disease. It concludes with a novel hypothesis that diversity observed in the *PRF1* gene may be an example of selective advantage that protects an individual from perforin-mediated pathology, such as BBB disruption.

## 1. Introduction

Diversity in the human immune response is important to effectively protect against extrinsic and intrinsic health threats. Two branches of immunity, innate and adaptive, are comprised of a variety of cells, each having a specific role in protecting the host from invading pathogens and malignancies. One branch of immunity specializes in recognizing and eliminating target cells through inducing apoptosis. These cells include CD8^+^ cytotoxic T lymphocytes (CTLs) and natural killer (NK) cells. CTLs recognize target cells through presentation of major histocompatibility complex class I (MHC I) molecules on the target cell surface. This relationship is often referred to as a lock and key, since the T cell receptor has fine specificity for MHCI: peptide complexes. In contrast, NK cells recognize target cells through loss of class I molecules, killer cell immunoglobulin receptors (KIRs), and killer cell lectin like receptors (KLRs). Although there is a difference in the target cell recognition process, once identified, both CTLs and NK cells execute similar effector function in order to remove the target cell. This is achieved through the release of cytotoxic granules containing perforin, granzymes, and granulysins, which work together to induce apoptosis in the target cells. This effector killing of target cells enables eradication of intracellular pathogens and malignancies.

**Figure 1:**
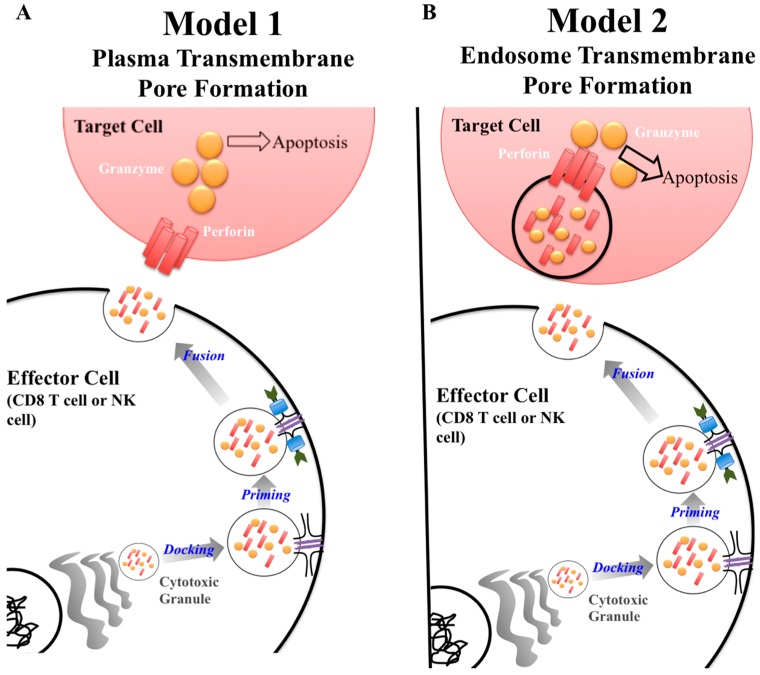
Current models of perforin granzyme synergy to induce apoptosis. There are two models to explain the synergistic effect between granzymes and perforin to induce apoptosis in a target cell. Both models are similar in regards to the process by which cytotoxic granules are produced by the effector cell. The difference lies at the target cell membrane surface. In Model 1, perforin forms a pore on the plasma membrane of the target cell allowing granzyme to be delivered to induce apoptosis (**A**). In model 2, granzyme and perforin are released from the cytotoxic granule, enter the target cell, and are then repackaged within the endosome. Perforin then forms a pore within the endosome, disrupting the membrane integrity, allowing granzyme to escape and induce apoptosis (**B**).

A key protein in the cytotoxic granule is perforin. Classically, perforin is known to form a pore in cell membranes, allowing passage of granzymes to induce apoptosis [1]. The exact synergy between perforin and granzymes is not fully understood [2]. Currently, there are two major models of how this process unfolds (Figure 1) [2,3,4]. The first model describes perforin forming a pore on the plasma membrane of the target cells allowing passage of granzyme into the cytoplasm inducing apoptosis (Figure 1A) [3]. The second model puts forward that perforin and granzymes are endocytosed by the target cell. Perforin then disrupts the endosome, which releases granzymes into the cytoplasm where it can interact with caspases resulting in apoptosis (Figure 1B) [3]. Although the mechanism of perforin-mediated cytotoxicity is still being defined, it is known that perforin is critical for protective immunity as demonstrated by gene knockout mice and human mutations having a deleterious phenotype [5,6,7,8].

In addition to its importance in cell-mediated immunity, perforin has been shown to play a critical role in immune-mediated blood–brain barrier (BBB) disruption [6]. The BBB ensures cells of the peripheral immune system, pathogens, and other blood-derived molecules do not enter the central nervous system (CNS). The BBB is comprised of a protective layer consisting of cerebral endothelial cells adhered by tight junctions, which sequester the CNS from the vasculature [9]. During injury or infection with pathogen, the BBB can be disrupted resulting in loss of tight junctions and increased permeability of the CNS [10,11,12,13]. The exact mechanism by which immune cells can contribute to BBB disruption is not fully understood and remains an active area of research. However, multiple studies have put forward a role for neutrophils, CD4^+^ T cells, CTLs, vascular endothelial growth factor [14] and various pro-inflammatory cytokines contributing to this particular pathology [14,15,16,17,18,19,20,21]. For the purposes of these studies, we focus on the role of CTLs and its effector molecule, perforin. In animal models, CTL induced BBB disruption has been determined to be perforin dependent [6]. However, it remains unclear whether the same process is applicable to human pathologies. Therefore, defining the dual role of perforin in protective immunity and CNS vascular permeability is an active area of research.

The first part of this review will focus on the evolution and diversity associated with the human perforin gene. Additionally, it will discuss perforin single nucleotide variants (SNVs) with relevance to human disease. It will conclude with a unifying hypothesis that diversity within the human perforin gene is an example of selective advantage that protects an individual from perforin-mediated pathology.

## 2. Perforin Evolution, Structure, and Function

### 2.1. Perforin Evolution

The perforin gene emerged prior to the divergence of *Chondrichthyes* and *Euteleostomi* about 500 million years ago [22]. The earliest organism recorded to have the perforin allele is *Gnathostomata*, or the jawed vertebrate. Earlier *Chordata* species do not present with evidence of a perforin gene, despite possessing traditional perforin wielding cell types [22]. Instead, it is suggested these species may possess macrophage expressed gene/protein 1 (MPEG1), a more primitive pore forming molecule which perforin is thought to have evolved. Along with perforin, other adaptive immune response mechanisms, including V(D)J recombination, emerged during this period [23]. These early genes are conserved throughout many vertebrates and are critical for an effective adaptive immune response. Many earlier species, including those of *Teleostei* and *Tetrapoda* family, have multiple copies of perforin gene [22]. In contrast, *Homo sapiens* and higher mammalian species have only one copy of the perforin allele. This reduced copy number does not diminish the importance of perforin in humans. Loss of perforin activity through mutation of the *PRF1* gene results in the lethal childhood disease familial hemophgocytic lymphohistiocytosis type 2 (FHL2), discussed later in this review [5].

### 2.2. Genetic and Proteome Organization of Perforin

**Figure 2:**
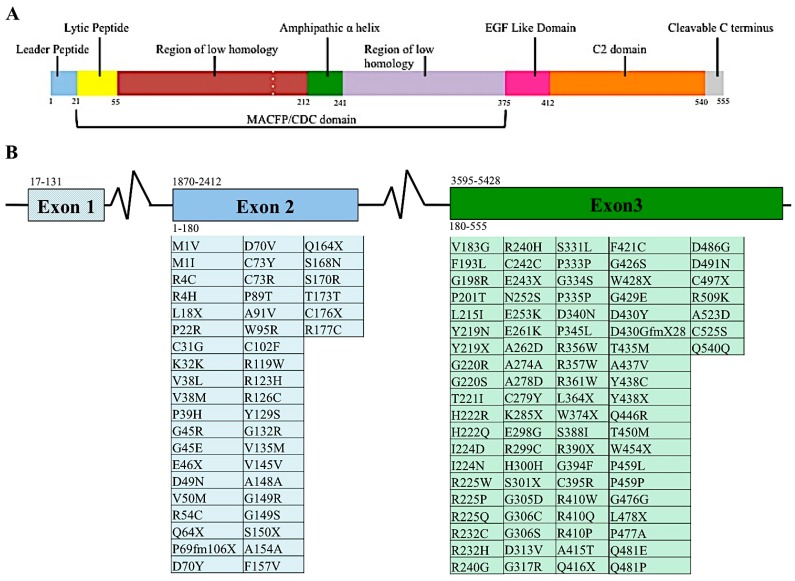
Proteomic and genomic organization of perforin. Linear representation of matured perforin protein illustrating the various domains and amino acids of this molecule (**A**). Diagram of *PRF1* indicating the nucleotide position of each exon, indicated by number above the diagram. Amino acids encoded by the respective exons are indicated by number below diagram. Mutations found in human populations, as reported in the literature, are listed below each corresponding exon (**B**). This list is not exhaustive of all *PRF1* SNVs identified.

The perforin gene locus, *PRF1*, consists of 3 exons. However, only exons 2 and 3 are translated to perforin protein [24,25]. When mature, perforin contains 555 amino acids, three domains, and has a mass of 67 kDa (Figure 2A). The most N-terminus domain of the perforin gene is the membrane attack complex perforin like/cholesterol dependent cytosylin (MACPF/CDC) domain. This domain assists in the polymerization of perforin. This domain serves a similar function in other proteins containing this region. Recently, it was suggested that the MACPF/CDC domain also facilitated granzyme B transfer through the perforin pore into the target cell’s cytoplasm [2]. This domain is present in numerous other human proteins, five of which are members of the innate immune response complement proteins: C6, C7, C8a, C8b, and C9. The last of these, C9, polymerizes after being recruited by the membrane attack complex (MAC), creating a pore in the cell’s plasma membrane [26]. Given their similar protein domains and function, C9 and perforin are considered homologous proteins [27]. The other proteins involved in the immune system containing that MACPF/CDC domain is MPEG1, the molecule perforin is believed to have evolved from [22]. Continuing towards the C-terminus of perforin are the epidermal growth factor (EGF) and calcium dependent C terminal (C2) domains (Figure 2A). These two domains come together to create a shelf-like structure [28]. The C terminus end of perforin binds to the lipid membrane in a calcium dependent manner. This in turn promotes perforin polymerization and formation of the pore that allows passage of granzymes to induce apoptosis [28].

### 2.3. Discovery of Perforin as a Key Component of Cytolytic Killing

In 1975, the “lethal hit” executed by CTLs was determined to be mediated by cytotoxic granules [29]. However, this concept was not fully accepted until 1983 when Podack and Dennert showed electron microscopy data of membrane lesions when NK cells engaged YAC-1 cells or erythrocytes. These membrane lesions were associated with cell cytotoxicity [30]. These data also supported the early work focusing on antibody dependent cellular toxicity and the complement component proteins pore formation [31]. Over the next decade, perforin, one of the proteins found in the cytotoxic granules, was isolated from mouse, rat, and human lymphocytes and NK cells [1]. Early work defined perforin as a pore forming molecule assisting in the induction of apoptosis. However, recent work suggests perforin may also serve as a recruitment molecule for monocytes and lymphocytes at the site of contact hypersensitivity (CHS) [32].

Once an effector cell releases cytotoxic granules, perforin inserts its most C-terminus region into a lipid membrane, in a calcium dependent manner. Then, through interactions in the MACPF/CDC domain, 19–24 perforin subunits polymerize, forming a pore [2,28]. Through the perforin pore, granzyme is selectively transported across the membrane. Once in the target cell’s cytoplasm, granzyme will induce apoptosis. As previously stated, there are two models defining which cellular membrane perforin inserts in to (Figure 1) [3]. The first model is the more traditional view of perforin in which the effector cell releases its content of the cytotoxic granule. Due to its close proximity, perforin forms a pore directly on the plasma membrane on the surface of the target cell (Figure 1A). In the second model, the effector cell releases the cytotoxic granule and the contents are endocytosed by the target cell. Once in the endosome, perforin forms a pore on the endosomal membrane granting granzyme access to the target cells cytoplasm (Figure 1B).

Unconventional roles for perforin beyond cytolytic killing have recently been identified [32,33,34,35,36]. Perforin may extend outside of the rather limited role of apoptotic induction and serve a purpose in down regulating the immune response. Perforin serves a non-apoptotic role during Herpes Simplex Virus 1 (HSV-1) infection of the neurons [33]. Studies have shown that HSV-1 infection is cleared in a CD8^+^ T cell and perforin dependent manner, yet neurons do not undergo apoptosis [33]. The mechanism in which perforin is involved in mediating the immune response is not yet determined but data suggests that perforin is involved in the antigen presentation process on dendritic cells as well as a role in a negative feedback loop to CD8^+^ T cells themselves [35,36]. As mentioned in brief earlier, during contact hypersensitivity perforin is suggested to serves as a recruitment molecule for additional lymphocytes to infiltrate at the site of hapten treated tissue [32]. These unconventional roles for perforin are surprising since there is currently no evidence of perforin acting on specific receptors, no defined mechanism of perforin acting as a monomer rather than polymerizing, and lack of evidence supporting the ability of perforin to interact with other proteins besides itself and other cytotoxic granule proteins, such as granzyme B. Additionally, there is no evidence that perforin pores have a function outside of target cell cytotoxicity.

### 2.4. Perforin Single Nucleotide Variants (SNVs)

Diversity among individuals is a hallmark feature of protective immunity, as clearly demonstrated by single nucleotide variants (SNVs) in the MHC class I and II molecules and large T cell and B cell repertoires. For these reasons, diversity at the perforin allele has been somewhat overshadowed by other branches of the immune system. Currently, over 450 SNVs, causing silent, frameshift or missense mutations, in the coding region of the perforin allele have been documented in the human population (Figure 2B) [5,37,38,39,40,41,42,43,44,45,46,47,48,49,50,51].

The vast majority of perforin mutations have not been investigated. Among the perforin mutations that have undergone structure and effector function analysis, multiple effects have been observed (Table 1) [40,41,50,52,53]. Perforin activity, defined as its ability to form pores and lyse red blood cells, is abolished, reduced or remains unaltered [40]. The most common perforin SNV, 272C>T, results in an alanine to valine mutation at amino acid residue 91 (A91V). This mutation is found in 4–17% of the population [54,55,56,57]. Catalytic studies using red blood cell lysis by single allele perforin indicate this mutation results in an almost 50% decrease in perforin activity [41]. A study investigating the effect of the A91V substitution suggests individuals heterozygous (WT/A91V) for this mutation will have approximately 75% total perforin activity. Meanwhile, those who are homozygous (A91V/A91V) have 50% perforin activity [41,52]. It is important to note that individuals with the A91V substitution will not necessarily succumb to disease, such as FHL 2. Overall, these individuals lead normal lives but are monitored for the onset of autoimmune disorders [58].

The nature and location of the perforin mutation plays a large role in this molecule’s activity. When a missense mutation lies within the C2 domain, the mutation tends to be deleterious. These mutations have the capacity to result in the lethal disease FHL 2 [40]. In contrast, mutations in the MACFP/CDC domain tend to be less deleterious to the protein with the exception of mutations that cause early truncation [40]. Furthermore, silent mutations that normally are thought to have minimal to no effect on protein function have significant recurrence in disease associated with decreased perforin activity. For example, in aplastic anemia, the silent mutation H300H (result of SNV 900C>T) is commonly linked with other non-deleterious mutations [44,59]. Since H300H does not affect perforin activity, its abnormally high presence in disease states remains unexplained and continues to be the topic of further research.

Certain geographical regions and ethnic groups present with more perforin mutations that affect activity [37,45,60]. Although there is much ambiguity in the origin of many perforin mutations, the 50delT SNV causing a truncation after leucine 18 (L18X), is most prevalent and believed to originate in African populations [46,61]. Additionally, the 50delT SNV has a high correlation with the previously mentioned polymorphism 900C>T, resulting in the H300H silent mutation. The origin of H300H has not been traced to a particular ethnic group at this time.

## 3. Human Disease Relevance of Perforin Single Nucleotide Variants

Perforin SNVs, specifically those that result in a mutation compromising perforin activity, are currently being investigated in the onset of a variety of diseases. In addition to FHL 2, type 1 diabetes mellitus, multiple sclerosis (MS), lymphomas, autoimmune lymphoproliferative syndrome (ALPS), and acquired aplastic anemia are all associated with perforin mutations [37,42,44,54,57,62]. A recurring mutation present in the aforementioned diseases is the A91V mutation, the most prevalent of the perforin mutations. In particular, the pairing of the A91V mutation with other potentially more severe mutations is being studied particularly in lymphomas and MS cases [42,44,57]. Based on these observations, a prevailing hypothesis is that non-deleterious perforin mutations are predisposing patients to disease by compromising the full potential of effector cells [57]. Not only could mutations affect the ability of the effector cells to lyse target cells, they could also affect the unconventional functions of perforin that are just recently being identified. The latter is just recently starting to be investigated.

### 3.1. Primary and Secondary Hemophagocytic Lymphohistiocytosis

Hemophagocytic Lymphohistiocytosis (HLH) is a rare disorder that causes high fever, splenomegaly, cytopenias, hepatitis, central nervous system dysfunction, and high morbidity. All are symptoms of pathological broad immune activation and uncontrolled inflammation [58,63]. HLH is categorized into two types; primary and secondary. Primary HLH has a genetic etiology while secondary HLH has an environmental etiology. Primary HLH is more commonly known as familial hemophagocytic lymphohistiocytosis (FHL). Although first described in 1952 by Farquhar and Claireaux, FHL had no known genetic etiology until 1999 [64]. Stepp et al. published the first known etiology of FHL, perforin dysfunction. Later, this perforin deficit would be classified as type 2 FHL (FHL 2) [5].

A single deleterious amino acid substitution can lead to FHL 2. However, multiple, compounding, non-deleterious substitutions can also lead to this disease. Currently, the precise pairings (if any) of non-deleterious substitutions resulting in FHL 2 are not known. Additionally, the maximum amount of perforin activity necessary to prevent FHL 2 remains undefined. However, one study suggests that 10–30% of CTLs need to be expressing fully active perforin in order to prevent FHL 2-like symptoms in a mouse model [65]. Once diagnosed, this disease has a mean life expectancy of two months without proper therapy. With proper therapy up to 55% of patients have a positive outcome [58].

Vaccinations, viral infections, autoimmune disorders, and cancers can trigger secondary HLH. Viral infections can range from various *herpeviridae* family members such as Epstein Barr virus (EBV), cytomegalovirus (CMV) and herpes simples virus (HSV), to *paramyxovirus* family such as avian influenza to retrovirus human immunodeficiency virus (HIV) [60,63,66]. Clinical signs of primary and secondary HLH are indistinguishable. For this reason, every patient presenting these symptoms is tested for a genetic deficit. If a genetic deficit is found, then the disease is primary HLH (or FHL). However, lacking a genetic deficit than the prognosis is secondary HLH [58].

### 3.2. FHL Prevalence

**Figure 3:**
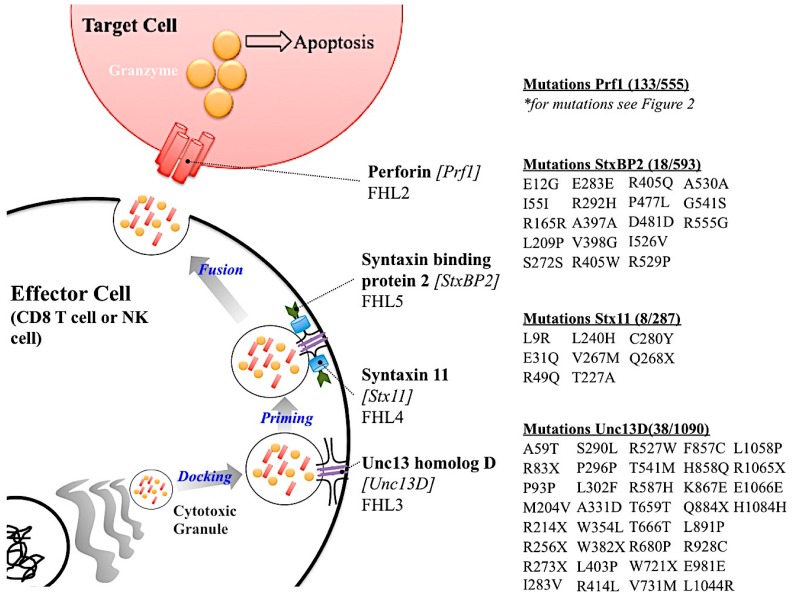
Summary of FHL genetic etiology. A schematic showing the role of the gene causative of FHL subtypes with the listed mutations. Genetic etiology for FHL 1 is not known and is therefore not reported in this Figure. Mutations listed were listed in primary literature. Please use the ExAC Browser database (http://exac.broadinstitute.org/) for more information regarding the mutations in these genes.

There are five subtypes of FHL (FHL 1–5) [39,60,67]. Types 2–5 have genes associated with them. The genetic etiology of FHL subtypes 2–5 are all part of the cytotoxic granule release pathway from effector cells (Figure 3). FHL 1 does not have an associated gene linked to its etiology. Since all other genes causing FHL are important in the cytotoxic granule release pathway from effector cells, it is possible that the unidentified gene for FHL 1 is also part of this pathway. Of the five types of FHL, FHL 2 is the most common, responsible for 20–40% of the cases worldwide (Table 2) [57]. FHL 2 is the result of mutations in the *PRF1* [5]. As discussed earlier there are over 450 SNVs in *PRF1*. Some, but not all, of these are associated with FHL 2. FHL 2 onset can be a result of single deleterious amino acid substitution or multiple non deleterious substitutions. FHL 2 is the only FHL subtype in which the known genetic etiology is a protein within the cytotoxic granule rather than a protein involved in the docking, priming or fusion process of granule release.

The second most common subtype of FHL is FHL 3, which is caused by deleterious mutations in the cytotoxic granule docking protein, Unc13 homolog D (Unc13D or Munc13-4), FHL 3 accounts for up to 20% of the cases (Table 2) [68]. A retrospective study conducted in Japan concluded that almost half the FHL cases reported were either FHL 2 or FHL 3 [45]. The other subtypes of FHL with genetic links, types 4 and 5, are caused by mutation in cytotoxic granule priming protein Syntaxin 11 (Stx11) and fusion protein Syntaxin binding protein 2 (StxBP2), respectively [49,69,70]. An interesting observation is that *PRF1* appears to have a higher amount of SNVs that are not related to disease compared to the other FHL-associated genes. However, this observation must be studied further. In sum, the high amount of SNVs in *PRF1* that are not associated with FHL 2, imply that perforin’s highly variant nature may serve a biological function in addition to contributing to human disease.

### 3.3. FHL 2 Incidence

Despite the high prevalence of perforin SNVs, diversity at this allele is not a contributing factor to FHL 2. This rare, autosomal recessive disorder has an incidence ranging 12–750 cases per 1 million children [45,49,71]. Males and females are afflicted with FHL 2 at a 1:1 ratio [49]. One reason a large range exists is a limited number of epidemiologic studies on FHL. Additionally, the ethnic regions in which studies were conducted also contribute to this difference in estimated prevalence. Sweden was reported to be on the low end of this range (12 cases per 1,000,000 children which correlate to 1 in 50,000 live births per year). In contrast, the prevalence of FHL 2 in Turkey is much higher with 7.5 cases per 10,000 children [49,71]. Meanwhile, FHL 2 incidence in Japan falls in the middle, with a reported incidence of 34.2 cases per 1,000,000 [45]. In total, the worldwide incidence is estimated to be approximately 1 in 50,000 live births (the result findings of Sweden]. The very high incidence of FHL in Turkey is commonly attributed to the high rate of consanguineous marriage, which is reported at about 30% frequency of marriages [72]. Whether consanguineous marriage is the sole cause of the high incidence of FHL 2 or if there are other selective pressures contributing needs to be further investigated. However, the higher incidence in Japan, as compared to Sweden, has not been explained. Therefore, FHL 2, similar to perforin SNVs, is associated with geographical location.

A question remains in why there is a large gap between the high prevalence of perforin SNVs in the human population and low incidence of the perforin deficiency disease FHL 2. Individuals who have reduced perforin activity caused by non-deleterious mutations, like A91V, appear to live healthy lives [46,73]. Upon further investigation, there will likely be additional non-deleterious mutations identified, which will reduce, but not abolish, perforin activity in otherwise healthy individuals. To potentially answer this question, we can revisit the observation that perforin mutations and incidence of FHL 2 varies greatly depending on geographical regions [45,46,61,71]. Interestingly, regions where FHL 2 is observed at a higher frequency may face environmental factors that select for reduced perforin activity.

A well-documented example of genetic heterogeneity being advantageous but retains an inherent risk of disease is sickle cell anemia. This disease causes abnormal hemoglobin resulting a crescent shaped red blood cells- is caused by mutations in the *HBB* [74]. When homozygous for the mutation, the disease presents itself, however if heterozygous for the gene, these people are resistant to malaria. The ancestry of this mutation is traced back to Africa, where there is a high prevalence of malaria [74]. The *HBB* mutation, among others polymorphisms at the hemoglobin allele, are advantageous to the population’s survival against malaria. It would therefore not be surprising if other genes, like perforin, would have a similar role in which an attenuated version provides greater survival. It is possible that FHL 2 is an unfortunate consequence of diversity in the perforin allele that can be advantageous to certain individuals. Supporting this hypothesis is the observation that perforin, along with providing protective immunity, contributes to significant pathology. Of major significance is the contribution of perforin to blood–brain barrier (BBB) disruption [6].

## 4. A Role for Perforin in Blood–Brain Barrier Disruption

Perforin SNVs have been studied in regard to causing deleterious perforinopathies such as FHL 2. However, if perforin diversity is studied in regards to enabling attenuation of immune mediated pathology, perforin SNVs can be associated with an evolutionary advantage. A major clue in how this could occur lies in the observation that perforin mediated disruption of the BBB in multiple animal models of disease [6,75]. BBB disruption in this case refers to the altering of cerebral endothelial cell tight junctions which results in increased CNS permeability; as perforin is not required for immune cell infiltration into the CNS [6,19]. The mechanism of how perforin causes BBB disruption is not fully understood. One possibility is perforin functions in its classical sense through inducing apoptosis of cells associated with the neurovascular unit. Another possibility is perforin is functioning through an unconventional means. The end result is compromised BBB integrity. While the mechanism by which perforin mediates BBB disruption is still being defined, the SNVs present in perforin allele that alter this molecule’s activity could affect its ability to mediate BBB disruption. This would explain why diversity in the human perforin allele is evolutionarily favorable in that it could temper immune mediated BBB disruption during infection with various pathogens.

### The Integrity and Disruption of the Blood–Brain Barrier

**Figure 4:**
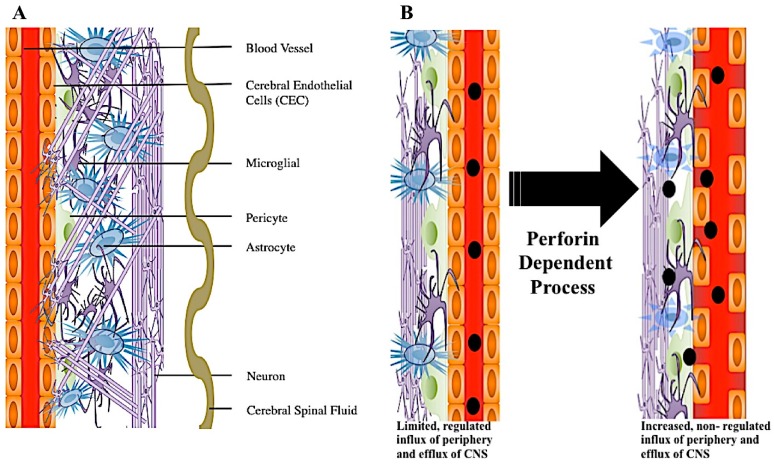
2D representation of perforin mediated disruption of the blood–brain barrier. Surrounding the blood vessel is a layer of endothelial cells held together by tight junctions. Encompassing the enodthelial cell layer are various brain cells comprising the neural vascular unit (NVU). NVU cell types include pericytes, astrocytes, microglia, and neurons. Additionally, sinuses run through this dense environment carrying cerebral spinal fluid (CSF) throughout the brain (**A**). During neuroinflammation, the blood–brain barrier can be disrupted through a perforin dependent process. During this process, CEC tight junctions become disorganized and CNS permeability occurs. Where there was once vascular integrity and sequestration of the CNS, there is now increased non-regulated influx of molecules from the blood (**B**).

The blood–brain barrier (BBB) is comprised of cerebral endothelial cells held together by tight junctions. Surrounding endothelial cells are astrocytes, pericytes, microglia, and innervated neurons [9] (Figure 4A). This highly specialized structure maintains separation from circulating blood and the neuronal network, thus protecting the central nervous system (CNS) from potential pathogens, while regulating pH and nutrient homeostasis [9,11]. Although small and lipophilic molecules may freely diffuse through the barrier, passage of large molecules is highly regulated [9]. Due to the protection the BBB provides to the brain, this organ was considered an immune privileged area. However, it is now recognized that the CNS has its own resident immune defenses, including microglia, which scavenge the brain and control potential infection [76,77]. Microglia are considered a component of innate immunity. However, following neurologic injury or infection, inflammatory cells from peripheral blood now readily infiltrate the CNS [78]. During this immune cell influx into the CNS, a variety of events could happen. One such event, cerebral endothelial cell tight junctions lose linear organization and this results in increased CNS vascular permeability [6]. This breaking down of cerebral endothelial cell tight junctions and increased permeability, allowing major influx of peripheral blood derived products into the CNS, is referred to as a major form of BBB disruption (Figure 4B).

BBB disruption is a feature of numerous neurological conditions including multiple sclerosis (MS), acute hemorrhagic leukoencephalitis (AHLE), epilepsy, traumatic brain injury, stroke, cerebral malaria [79], and viral hemorrhagic fevers (VHFs) [10,79,80,81,82,83,84,85,86,87,88,89,90,91,92,93,94,95,96,97,98]. In these scenarios, the brain experiences an influx of cells such as neutrophils, T cells and other circulating monocytes. In many cases, BBB disruption is pathogenic. However, in certain scenarios it is even fatal. VHF’s not only cause BBB disruption but also mount a large inflammatory response [99]. The viral antigens trigger the immune cells, and although acting to clear the infection, in this setting the cost out weighs the benefit and the host could experience severe brain damage or even death [75,98].

The main effector cell necessary to cause BBB disruption is currently under debate. In models using the arenavirus Lymphocytic choriomeningitis virus (LCMV), a negative single stranded RNA, enveloped virus, neutrophil infiltration appears to be the initial step during BBB disruption and is believed to play a key role in this process [16]. However, in other models using murine pircornavirus, Theiler’s murine encephalomyelitis virus (TMEV), a positive single stranded, naked, RNA virus, BBB disruption occurs independent of neutrophils [17,18,100,101,102]. In contrast, BBB disruption occurs in an antigen dependent and perforin dependent manner. In other models of pathogen-associated BBB disruption, such cerebral malaria, perforin and CD8^+^ T cells have been documented to promote BBB [19,98,103]. Discerning the critical immune factors that contribute to BBB disruption is critical in better understanding the mechanism by with this potentially fatal pathology can occur.

**Figure 5:**
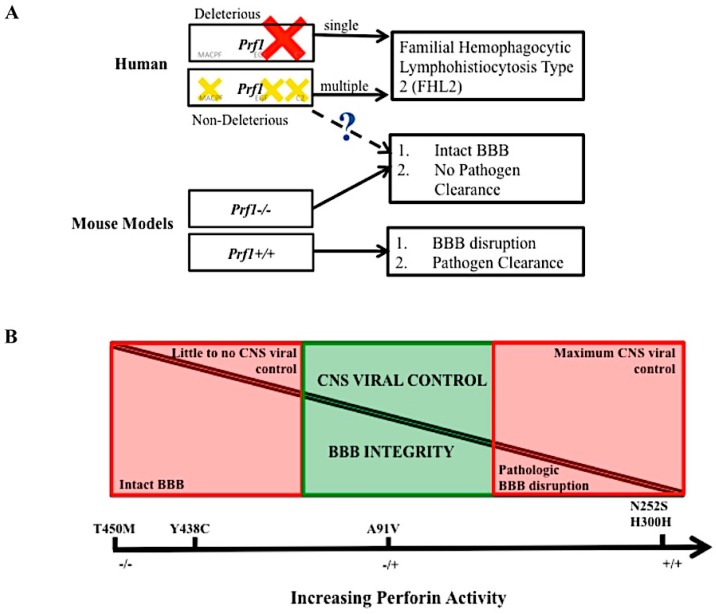
The balance of perforin expression may be advantageous to an individual’s fitness. Hypothetical model of perforin’s contribution to pathogen clearance and vascular permeability. Loss of perforin activity in the human due to single deleterious mutation (**red cross**) or multiple, compounding mutations (**yellow crosses**) leads to disease FHL 2. In the mouse model, mice lacking perforin cannot clear virus in the CNS but do not experience BBB disruption. Perforin competent mice can clear virus in the CNS but experience lethal BBB disruption. The dashed line and question mark indicate a level of perforin, found in the human population that may mediate BBB disruption but still allow for CNS pathogen control (**A**). At full perforin activity, CNS viral control is possible, however there is pathologic BBB disruption. At no perforin expression, pathologic BBB disruption is not present, but there is little of CNS viral infection. Non deleterious perforin SNVs that decrease but do not abolish perforin’s activity may provide a balance between these two pathologies (**B**). The five SNVs listed are purely there as a frame of reference, we do not conclude that these exact polymorphisms play a role in this balance.

An analysis of how virus specific CD8^+^ T cells use perforin to disrupt the BBB can be performed using Peptide Induced Fatal Syndrome (PIFS) model [100,102]. PIFS is induced in TMEV infected mice. Following intracranial infection with TMEV, viral specific CD8^+^ T cells infiltrate the brain to eliminate the infection. During the peak of this response at seven days post infection, 50–70% of brain infiltrating CD8^+^ T cells recognize the immunodominant VP2_121–130_ peptide presented in the context of the D^b^ class I molecule [104]. At sevens day post infection, VP2_121–130_ peptide is intravenously administered to C57BL/6 mice. D^b^: VP2_121–130_ epitope specific CD8^+^ T cells respond resulting in compromised integrity of the BBB [6]. Ultimately, this heightened immune response and ensuing BBB disruption results in mice to become moribund within 12–18 hour post systemic antigen exposure [6,100,102]. Mice lacking perforin (perforin−/−) are resistant to PIFS despite having equivalent infiltration of D^b^:VP2_121–130_ epitope specific CD8^+^ T cells into the brain [6]. Determining how perforin contributes to BBB in the PIFS model, as well as in experimental cerebral malaria, continues to be an active area of research. Given the newly identified non-cytolytic properties of perforin, it is possible this molecule is functioning through an unconventional mechanism to mediate BBB disruption. Furthermore, the various human mutations identified could be affecting perforin activity and function in this process. We put forward that the large number of SNVs found in the human population, suggest that a partially active form of perforin may retain beneficial cytotoxic killing to control pathogen infection, yet at times also temper the CD8^+^ T cell response to reduce pathologic BBB disruption and mortality [Figure 5].

## 5. Conclusions and Remaining Questions

Since its discovery in 1975, perforin has been defined as a pore forming effector molecule involved in cytolytic killing [1,29]. However, recent studies investigating perforin’s effect in disease models require a reevaluation of this molecule’s role in disease. In addition to forming a pore on a lipid membrane of target cells to allow passage of granzyme and induce apoptosis, perforin may also have some immune regulatory effects [32,35,36]. Additionally, the human perforin allele is variant. This is surprising considering the other homologous pore forming molecule of the immune system, complement component 9 (C9) has less SNVs despite its similar size and function as perforin [51,105]. Furthermore, the high frequency of perforin SNVs in the human population does not match up with the diseases associated with loss of perforin function.

The SNVs in perforin have a range of effects which include: (1) no effect; (2) decreased activity; (3) early truncation; or (4) complete loss of function and/or misfolding [40,41,50,52,55]. Multiple perforin SNVs, resulting in multiple substitutions, can result in complete loss of perforin activity. Losing perforin activity results in a rare, lethal childhood disease FHL 2 [5]. Other studies indicate that certain perforin SNVs are associated with type 1 diabetes mellitus, multiple sclerosis, lymphomas, autoimmune lymphoproliferatie syndrome (ALPS) and acquired aplastic anemia [37,42,44,54,57,62]. Perforin also mediates BBB disruption as demonstrated in studies using experimental cerebral malaria and the PIFS model [6,75].

Overall, many questions remain. How does perforin contribute to diseases such as FHL? What is the significance of perforin SNVs in humans, given that this molecule’s function has been conserved for millions of year across many species? Is immune-mediated pathology, including BBB disruption, a bystander effect of inflammation designed to protect us from pathogens? Is variability in the human perforin allele advantageous in protecting against the wide variety of pathogens humans encounter? Diversity is a hallmark feature of the human immune system, which includes the highly polymorphic MHC class I and II molecules and vast repertoires of T cell receptor and antibody responses. In each of these examples, the variant nature is advantageous to the population as a whole. One could therefore conclude perforin may serve an analogous role.

## Figures and Tables

**Table 1:** List of selected *PRF1* single nucleotide variants (SNVs). Polymorphisms shown have had activity tested by other research groups. For each SNV listed, the resulting protein change and functional consequence is listed. All listed SNVs have been identified in FHL 2* patients, however this list is not exhaustive.

DNA Change	Protein Change	Domain Location	Functional Consequence	Reference
272C>T	A91V	Region of Low homology I	Decreased activity	38
548T>G	V183G	Region of Low homology I	Activity maintained	5
577T>C	F193L	Region of Low homology I	Conformational change, protein degraded	52
662C>T	T221I	Amphipathic α-helix	Conformational change, protein degraded	52
673C>T	R225W	Amphipathic α-helix	Activity abolished	52
695G>A	R232H	Amphipathic α-helix	Decreased activity	52
755A>G	N252S	Region of Low homology II	Activity maintained	46
822C>T	A274A	Region of Low homology II	Activity maintained	46
836G>A	C279Y	Region of Low homology II	Activity abolished	5
900C>T	H300H	Region of Low homology II	Activity maintained	46
1122G>A	W374X	Region of Low homology II	Activity abolished	5
1228C>T	R410W	EGF like domain	Conformational change, protein degraded	52
1229G>C	R410P	EGF like domain	Conformational change, protein degraded	52
1286G>A	G429E	C2 Domain	Activity abolished	52
1304C>T	T435M	C2 Domain	Activity abolished	50
1313A>G	Y438C	C2 Domain	Matures improperly, some activity present	50

* familial hemophagocytic lymphohistiocytosis type 2.

**Table 2:** Summary of FHL Incidence. Listed are each FHL subtype, the percentage of particular subtypes in respect to all FHL cases, the etiological gene, and the gene size. Amount of mutations in coding region of gene was calculated using mutations found in Leiden Open Variation Dataase, NCBI SNP database, and primary literature.

FHL Type	Percentage of FHL Types for All FHL Cases	Gene	Protein Size (Amino Acids)	Number of Mutations in Coding Region of Gene	Mutation Rate (per 100 Amino Accids)
FHL 1	4 cases total	Unknown	Unknown	Unknown	Unknown
FHL 2	20–40% (>50% in African American families)	*PRF1*	555	133	23.9
FHL 3	20–30%	*UNC13D*	1090	38	3.5
FHL 4	20% in Turkish families	*STX11*	287	8	2.7
FHL 5	15–20%	*STXBP2*	593	18	3.0

## Abbreviations

FHL 2	Familial hemophagocytic lymphohistiocytosis type 2
BBB	Blood–brain barrier
SNV	Single nucleotide variant
NK	Natural Killer
CTL	CD8^+^ cytotoxic T lymphocytes
MHC I	Major histocompatibility complex class I
KIR	Killer cell immunoglobulin receptor
KLR	Killer cell lectin like receptor
CNS	Central nervous system
VEGF	Vascular endothelial growth factor
MPEG1	Macrophage expressed gene/protein 1
MACPF/CDC	Membrane attach complex perforin like/cholesteral dependent cytosylin
MAC	Membrane attack complex
EGF	Epidermal growth factor
MS	Multiple sclerosis
ALPS	Autoimmune lymphoproliferative syndrome
HLH	Hemophagocytic Lymphohistiocytosis
EBV	Epstein Barr Virus
CMV	Cytomegalovirus
Stx11	Syntaxin 11
StxBP2	Syntaxin binding protein 2
NVU	Neurovascular unit
CSF	Cerebral spinal fluid
CEC	Cerebral endothelial cell
TMEV	Theiler’s Murine encephalomyelitis virus
PIFS	Peptide induced fatal syndrome
ECM	Experimental cerebral malaria
CM	Cerebral malaria
VHF	Viral hemorrhagic fever
LCMV	Lymphocytic choriomeningitis virus
